# Supplementary figures and images for: Coevolution of Eukaryote-like Vps4 and ESCRT-III Subunits in the Asgard Archaea
Source: mBio. 2020 May 19;11(3):e00417-20. doi: 10.1128/mBio.00417-20 (PMC7240154; doi:10.1128/mBio.00417-20)

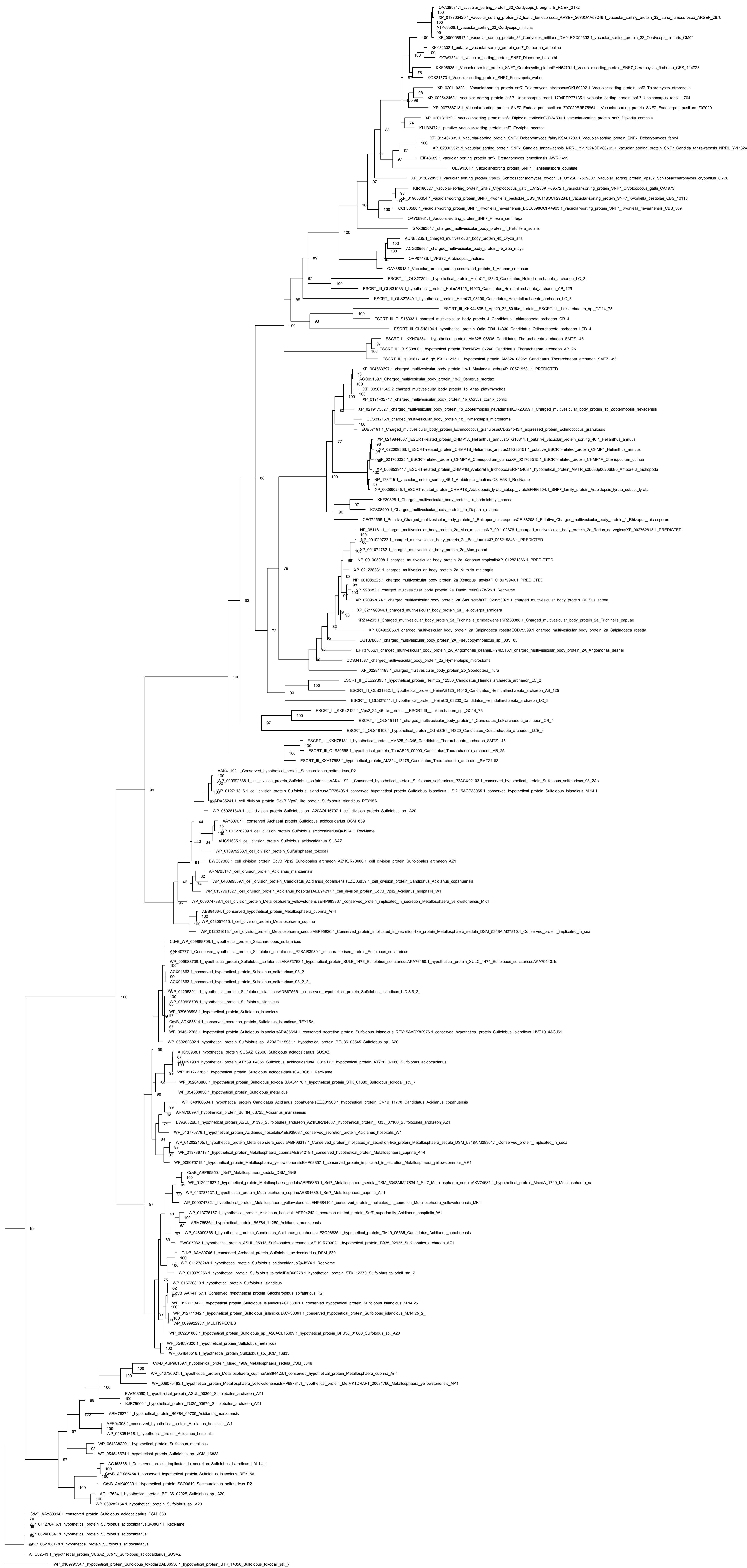

Supplement: FIG S1 [file mBio.00417-20-sf001.pdf]

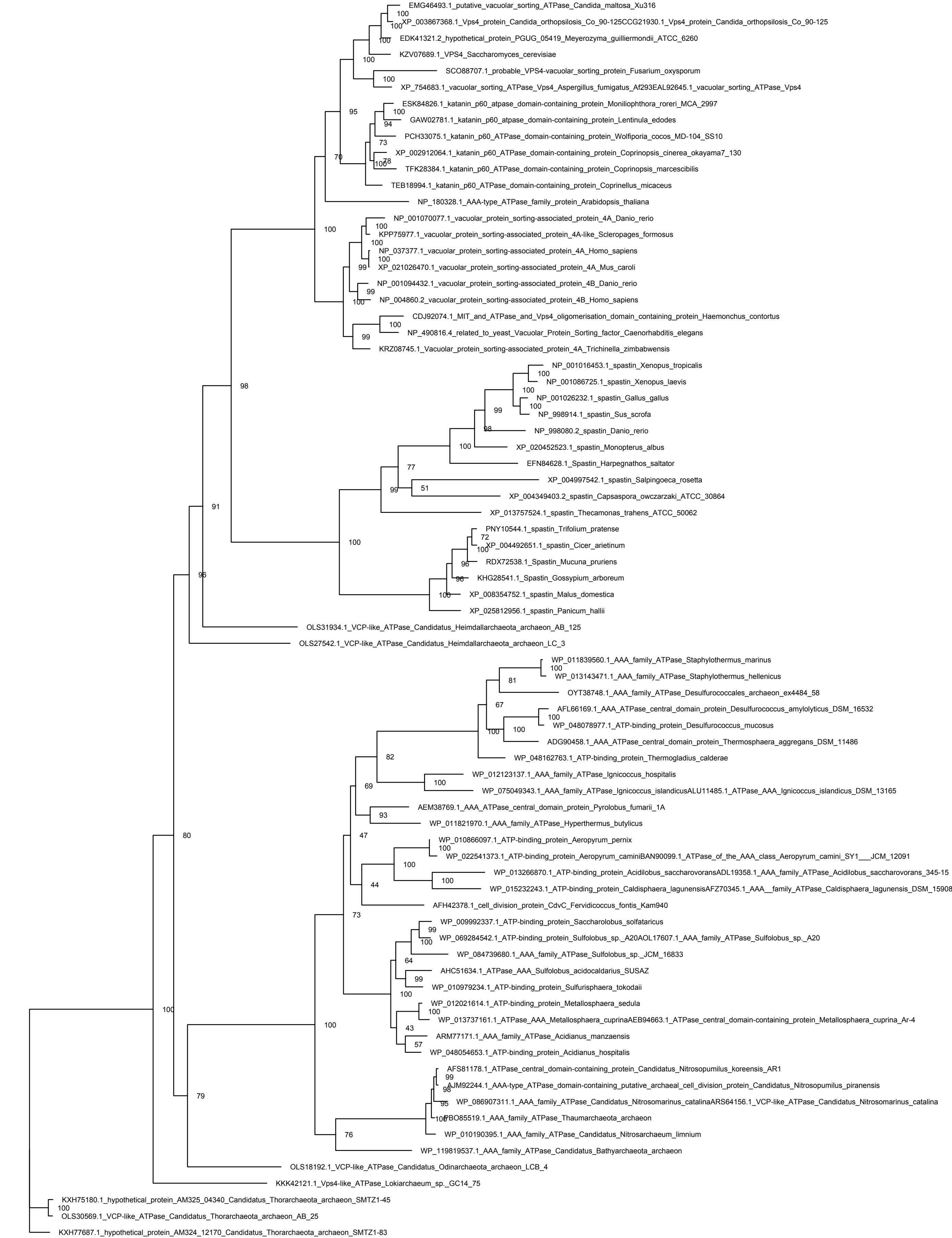

Supplement: FIG S2 [file mBio.00417-20-sf002.pdf]

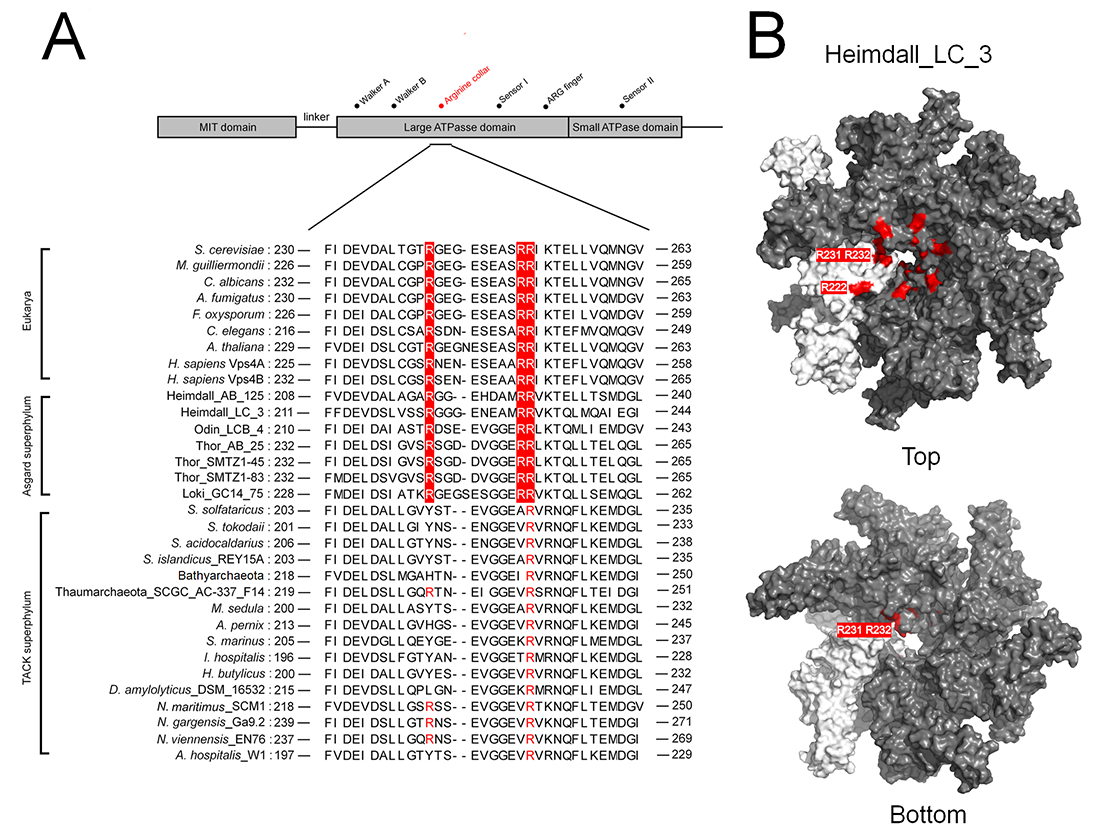

Supplement: FIG S3 [file mBio.00417-20-sf003.tif]

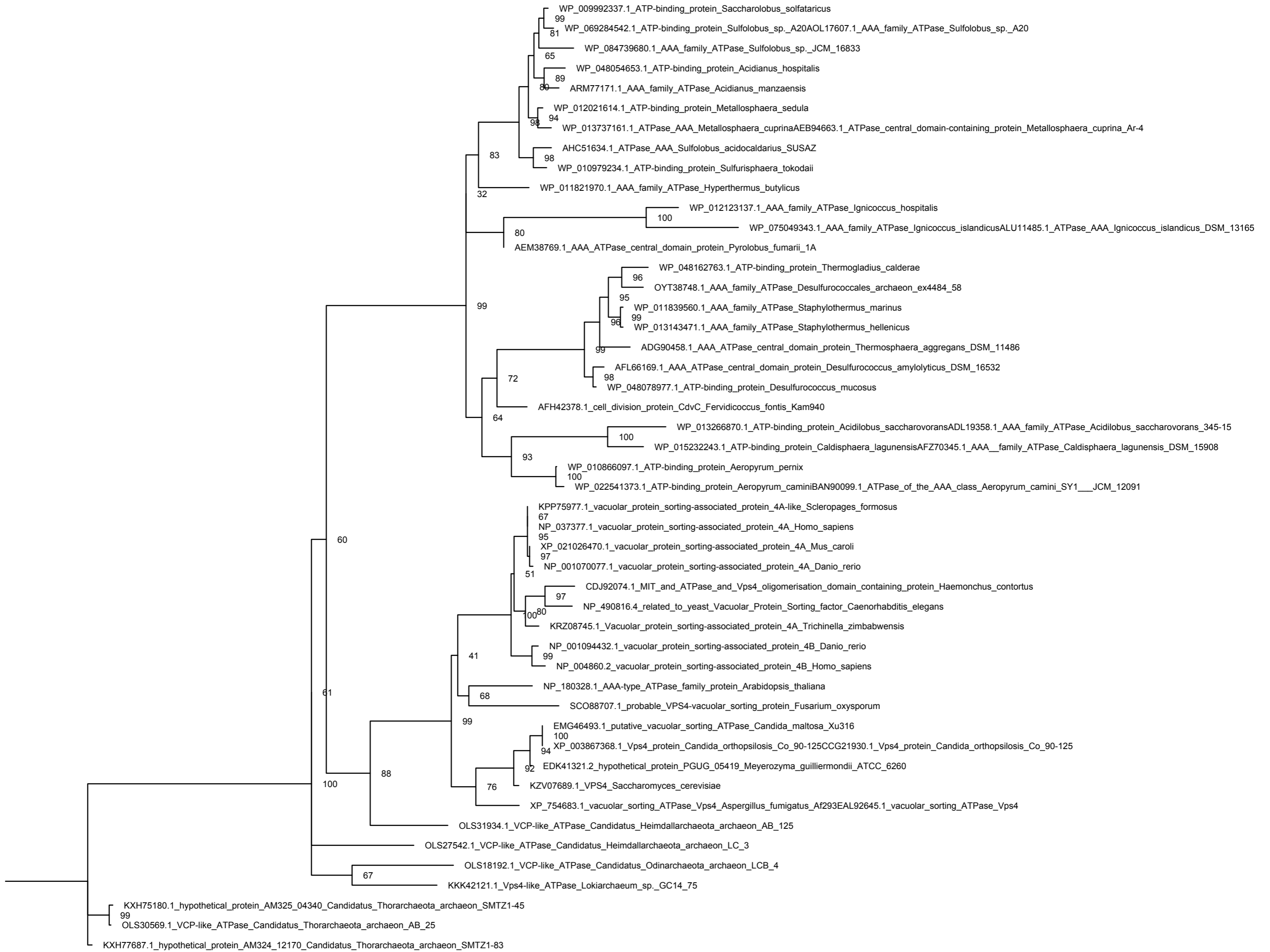

Supplement: FIG S4 [file mBio.00417-20-sf004.pdf]

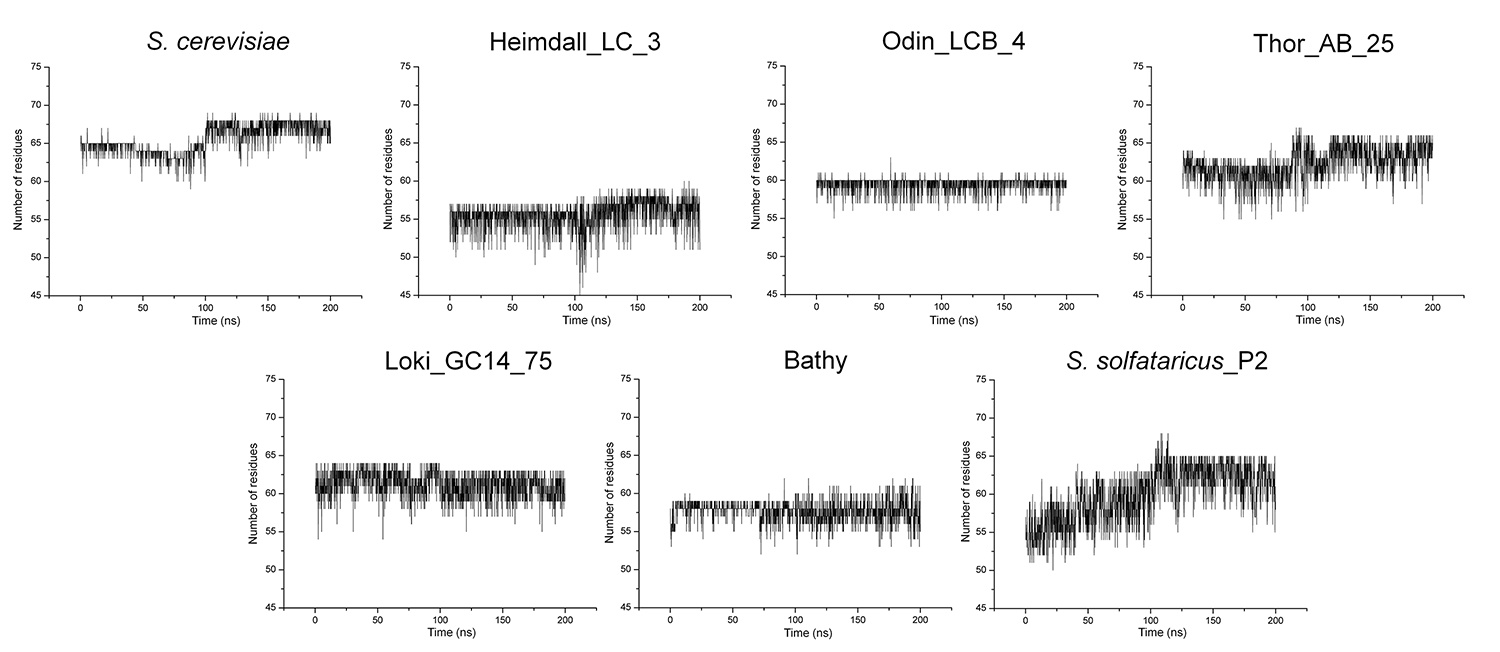

Supplement: FIG S5 [file mBio.00417-20-sf005.tif]

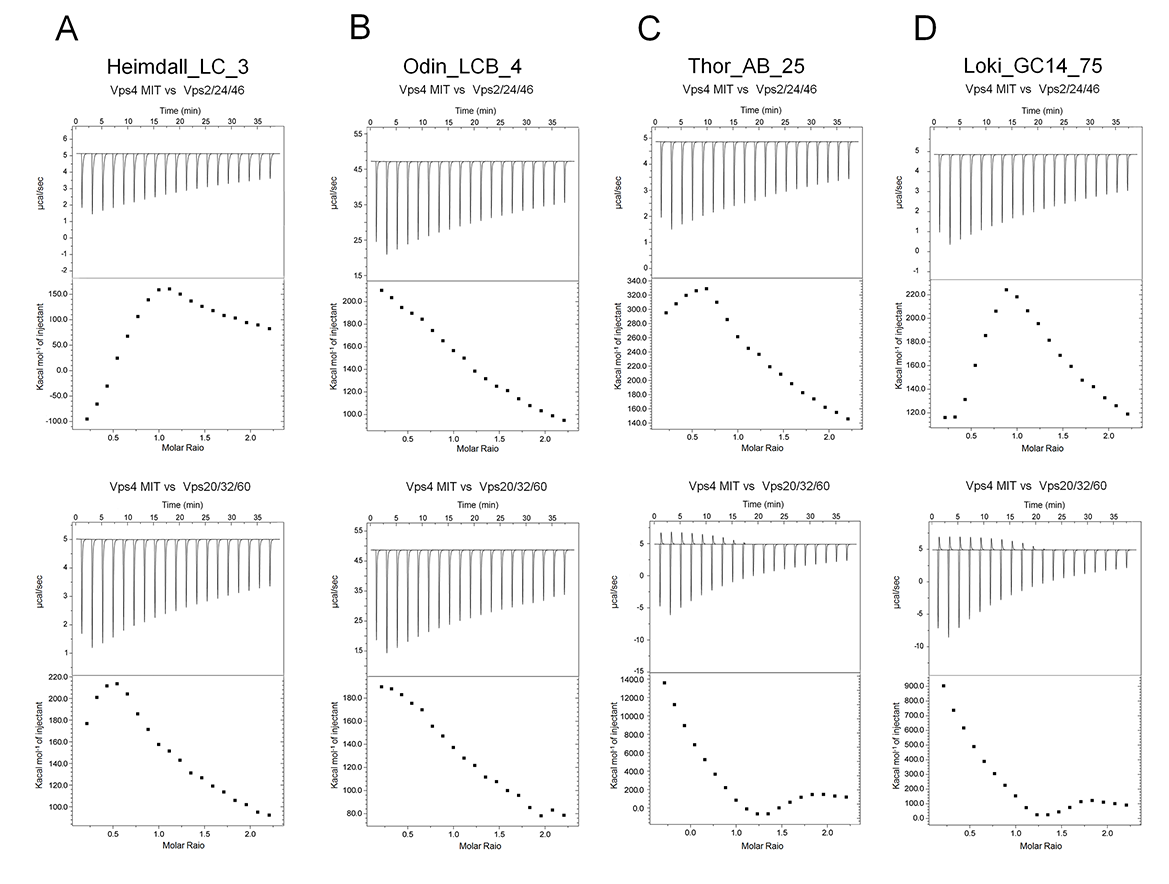

Supplement: FIG S6 [file mBio.00417-20-sf006.tif]
