# Supplementary material for: Coevolution of Eukaryote-like Vps4 and ESCRT-III Subunits in the Asgard Archaea
Source: mBio. 2020 May 19;11(3):e00417-20. doi: 10.1128/mBio.00417-20 (PMC7240154; doi:10.1128/mBio.00417-20)
Supplement: TABLE S1 [file mBio.00417-20-st001.docx]

**TABLE S1. Summary of proteins used in this study.**

| **Species** | **Protein** | **NCBI accession** |
| --- | --- | --- |
| Thorarchaeota_SMTZ1-83 (Thor_SMTZ1-83) | Vps2/24/46 | KXH77688.1 |
|  | Vps20/32/60 | KXH71213.1 |
|  | Vps4 | KXH77687.1 |
| Thorarchaeota_AB_25 (Thor_AB_25) | Vps2/24/46 | OLS30568.1 |
|  | Vps20/32/60 | OLS30800.1 |
|  | Vps4 | OLS30569.1 |
| Thorarchaeota_SMTZ1-45 (Thor_SMTZ1-45) | Vps2/24/46 | KXH75181.1 |
|  | Vps20/32/60 | KXH70284.1 |
|  | Vps4 | KXH75180.1 |
| Odinarchaeota_LCB_4 (Odin_LCB_4) | Vps2/24/46 | OLS18193.1 |
|  | Vps20/32/60 | OLS18194.1 |
|  | Vps4 | OLS18192.1 |
| Lokiarchaeota_CR_4 (Loki_CR_4) | Vps2/24/46 | OLS15111.1 |
|  | Vps20/32/60 | OLS16333.1 |
| Lokiarchaeum_GC14_75 (Loki_GC14_75) | Vps2/24/46 | KKK42122.1 |
|  | Vps20/32/60 | KKK44605.1 |
|  | Vps4 | KKK42121.1 |
| Heimdallarchaeota_LC_3 (Heimdall_LC_3) | Vps2/24/46 | OLS27541.1 |
|  | Vps20/32/60 | OLS27540.1 |
|  | Vps4 | OLS27542.1 |
| Heimdallarchaeota_AB_125 (Heimdall_AB_125) | Vps2/24/46 | OLS31932.1 |
|  | Vps20/32/60 | OLS31933.1 |
|  | Vps4 | OLS31934.1 |
| Heimdallarchaeota_LC_2 (Heimdall_LC_2) | Vps2/24/46 | OLS27395.1 |
|  | Vps20/32/60 | OLS27394.1 |
| *Saccharomyces cerevisiae* (*S. cerevisiae*) | Vps2 | NP_012924.2 |
|  | Vps24 | CAA81876.1 |
|  | Vps46 | KZV09977.1 |
|  | Vps20 | KZV08919.1 |
|  | Vfa1 | KZV11896.1 |
|  | Vps32 | NP_013125.1 |
|  | Vps4 | KZV07689.1 |
| *Homo sapiens* (*H. sapiens*) | Vps2 | NP_055268.1 |
|  | CHMP7 | NP_689485.1 |
|  | Vps20 | XP_005257725.1 |
|  | Vps4A | NP_037377.1 |
|  | Vps4B | NP_004860.2 |
| *Arabidopsis thaliana* (*A. thaliana*) | Vps20 | NP_196488.1 |
|  | Vps4 | NP_180328.1 |
| *Anopheles gambiae* (*A. gambiae*) | Vps20 | EAA11586.4 |
| *Cordyceps fumosorosea* (*C. fumosorosea*) | Vps32 | XP_018701501.1 |
| *Calocera cornea* (*C. cornea*) | Vps2 | KZT58940.1 |
| *Cordyceps confragosa* (*C. confragosa*) | Vps32 | OAA70839.1 |
| *Schizosaccharomyces pombe* (*S. pombe*) | Vps20 | NP_596691.1 |
| *Aspergillus tanneri* (*A. tanneri*) | Vps2 | KAA8652701.1 |
| *Beauveria bassiana* (*B. bassiana*) | Vps32 | XP_008596828.1 |
| *Zygosaccharomyces parabailii* (*Z. parabailii*) | Vps46 | AQZ11685 |
| *Rhizoctonia solani* (*R. solani*) | Vps2 | CUA73503.1 |
| *Kluyveromyces marxianus* (*K. marxianus*) | Vps46 | QGN15989.1 |
| *Hyphopichia burtonii* (*H. burtonii*) | Vps46 | XP_020077182.1 |
| *Suhomyces tanzawaensis* (*S. tanzawaensis*) | Vps46 | XP_020063767.1 |
| *Phialocephala scopiformis* (*P. scopiformis*) | Vps2 | XP_018076086.1 |
| *Mus musculus* (*M. musculus*) | Vps20 | NP_001078967.1 |
| *Meyerozyma guilliermondii* (*M. guilliermondii*) | Vps4 | EDK41321.2 |
| *Candida albicans* (*C. albicans*) | Vps4 | XP_720644.1 |
| *Aspergillus fumigatus* (*A. fumigatus*) | Vps4 | EDP52808.1 |
| *Fusarium oxysporum* (*F. oxysporum*) | Vps4 | SCO88707.1 |
| *Nitrosarchaeum limnium*_BG20 (*N. limnium*_BG20) | CdvB | WP_010191799.1 |
| *Nitrosopumilus koreensis*_AR1 (*N. koreensis*_AR1) | CdvB | WP_014963169.1 |
| Bathyarchaeota_BE326-BA-RLH  (Bathy_BE326-BA-RLH) | CdvB | WP_119818818.1 |
|  | CdvC | WP_119819537.1 |
| *Sulfolobus acidocaldarius*_DSM_639  (*S. acidocaldarius*_DSM_639) | CdvB | AAY80707.1 |
|  | CdvC | ADX85242.1 |
| *Sulfolobus islandicus*_REY15A  (*S. islandicus*_REY15A) | CdvB | ADX85241.1 |
| *Saccharolobus solfataricus*_P2 (*S. solfataricus*_P2) | CdvB | AAK41192.1 |
| *Sulfurisphaera tokodaii* (*S. tokodaii*) | CdvB | WP_010979233.1 |
|  | CdvC | WP_010979234.1 |
| *Sulfolobus acidocaldarius* (*S. acidocaldarius*) | CdvC | WP_011278208.1 |
| *Saccharolobus solfataricus* (*S. solfataricus*) | CdvC | WP_009992337.1 |
| Thaumarchaeota_SCGC_AC-337_F14 | CdvC | WP_119819537.1 |
| *Metallosphaera sedula* (*M*. *sedula*) | CdvC | WP_012021614.1 |
| *Aeropyrum pernix* (*A. pernix*) | CdvC | WP_010866097.1 |
| *Staphylothermus marinus* (*S. marinus*) | CdvC | WP_011839560.1 |
| *Ignicoccus hospitalis* (*I. hospitalis*) | CdvC | WP_012123137.1 |
| *Hyperthermus butylicus* (*H. butylicus*) | CdvC | WP_011821970.1 |
| *Nitrosopumilus maritimus*_SCM1  (*N. maritimus*_SCM1) | CdvC | ZP_02024063.1 |
| *Desulfurococcus amylolyticus*_DSM_16532  (*D. amylolyticus*_DSM_16532) | CdvC | AFL66169.1 |
| *Nitrososphaera gargensis*_Ga9.2 (*N. gargensis*_Ga9.2) | CdvC | AFU57509.1 |
| *Nitrososphaera viennensis*_EN76 (*N. viennensis*_EN76) | CdvC | AIC16822.1 |
| *Acidianus hospitalis*_W1 (*A. hospitalis*_W1) | CdvC | AEE94218.1 |
