# Supplementary material for: Coevolution of Eukaryote-like Vps4 and ESCRT-III Subunits in the Asgard Archaea
Source: mBio. 2020 May 19;11(3):e00417-20. doi: 10.1128/mBio.00417-20 (PMC7240154; doi:10.1128/mBio.00417-20)
Supplement: TABLE S2 [file mBio.00417-20-st002.docx]

**TABLE S2. The predicted binding free energies between Vps4 and ESCRT-III subunits (Vps2/24/46 (A), and Vps20/32/60 (B)).**

**TABLE S1A. The predicted binding free energies between Vps4 and Vps2/24/46.**

|  | ΔE_ele_ | |  | ΔE_VDW_ | |  | ΔG_np_ | |  | ΔG_ele_ | |  | ΔG_bind_ | |
| --- | --- | --- | --- | --- | --- | --- | --- | --- | --- | --- | --- | --- | --- | --- |
|  | X | SD |  | X | SD |  | X | SD |  | X | SD |  | X | SD |
| *S. cerevisiae* | -67.10 | 4.02 |  | -148.94 | 7.18 |  | -25.41 | 1.31 |  | 158.47 | 6.78 |  | -82.98 | 11.25 |
| Heimdall_LC_3 | -437.37 | 93.19 |  | -43.62 | 3.26 |  | -9.01 | 1.03 |  | 450.96 | 90.31 |  | -39.02 | 7.17 |
| Odin_LCB_4 | -657.37 | 73.31 |  | -94.01 | 18.59 |  | -16.15 | 3.29 |  | 705.67 | 97.14 |  | -61.85 | 1.95 |
| Thor_AB_25 | -415.72 | 77.36 |  | -131.17 | 15.90 |  | -22.24 | 2.66 |  | 497.31 | 94.15 |  | -71.81 | 1.78 |
| Loki_GC14_75 | -1955.85 | 80.41 |  | -84.49 | 7.21 |  | -16.87 | 1.73 |  | 1984.95 | 88.06 |  | -72.24 | 1.29 |

**TABLE S1B. The predicted binding free energies between Vps4 and Vps20/32/60.**

|  | ΔE_ele_ | |  | ΔE_vdw_ | |  | ΔG_np_ | |  | ΔG_ele_ | |  | ΔG_bind_ | |
| --- | --- | --- | --- | --- | --- | --- | --- | --- | --- | --- | --- | --- | --- | --- |
|  | X | SD |  | X | SD |  | X | SD |  | X | SD |  | X | SD |
| *S. cerevisiae* | -577.69 | 247.47 |  | -108.29 | 3.46 |  | -19.85 | 1.51 |  | 617.52 | 249.45 |  | -88.30 | 2.98 |
| Heimdall_LC_3 | -852.54 | 283.90 |  | -95.14 | 12.95 |  | -10.29 | 13.87 |  | 886.54 | 281.40 |  | -81.43 | 15.72 |
| Odin_LCB_4 | -663.36 | 169.52 |  | -81.52 | 12.69 |  | -14.55 | 2.38 |  | 709.89 | 173.50 |  | -49.53 | 11.09 |
| Thor_AB_25 | 210.54 | 137.95 |  | -181.64 | 21.34 |  | -29.63 | 3.17 |  | -119.24 | 138.68 |  | -119.97 | 25.24 |
| Loki_GC14_75 | -2531.29 | 4.57 |  | -94.88 | 7.88 |  | -17.26 | 0.73 |  | 2554.54 | 11.04 |  | -88.89 | 2.14 |

The binding free energy is approximately expressed as the sum of molecular mechanics (ΔE_ele_: electrostatic energy in the gas phase, ΔE_vdw_: van der Waals energy) and solvation energies (ΔG_np_: nonpolar solvation energy ΔG_ele_: polar solvation energy). Data are shown as the X (mean value) and SD (standard deviation). *S. cerevisiae*: *Saccharomyces cerevisiae*; Heimdall_LC_3: Heimdallarchaeota_LC_3; Odin_LCB_4: Odinarchaeota_LCB_4; Thor_AB_25: Thorarchaeota_AB_25; Loki_GC14_75: Lokiarchaeum_GC14_75.
