# Supplementary material for: Coevolution of Eukaryote-like Vps4 and ESCRT-III Subunits in the Asgard Archaea
Source: mBio. 2020 May 19;11(3):e00417-20. doi: 10.1128/mBio.00417-20 (PMC7240154; doi:10.1128/mBio.00417-20)
Supplement: TABLE S3 [file mBio.00417-20-st003.docx]

**TABLE S3. The dominant amino acid residues of Vps4 Microtubule Interacting and Transport domain involved in binding with Vps2/24/46 are listed.**

| *S. cerevisiae* | | |  | Heimdall_LC_3 | | |  | Odin_LCB_4 | | |  | Thor_AB_25 | | |  | Loki_GC14_75 | | |
| --- | --- | --- | --- | --- | --- | --- | --- | --- | --- | --- | --- | --- | --- | --- | --- | --- | --- | --- |
| Residue | Total | SD |  | Residue | Total | SD |  | Residue | Total | SD |  | Residue | Total | SD |  | Residue | Total | SD |
| K16 | -3.48 | 1.31 |  | Q43 | -1.84 | 0.66 |  | V20 | -0.64 | 0.59 |  | M24 | -2.17 | 1.12 |  | S2 | -1.62 | 1.73 |
| L20 | -1.14 | 0.27 |  | F46 | -1.54 | 0.32 |  | K53 | -3.02 | 1.61 |  | Q30 | -1.53 | 1.96 |  | S3 | -2.60 | 1.49 |
| Y30 | -1.63 | 0.78 |  | R47 | -13.34 | 1.49 |  | I57 | -1.29 | 0.74 |  | L33 | -1.4 | 0.40 |  | K7 | -3.86 | 2.47 |
| T31 | -2.97 | 0.61 |  | K55 | -4.84 | 1.71 |  | K60 | -2.64 | 2.29 |  | K34 | -2.52 | 1.50 |  | L8 | -2.80 | 0.46 |
| A32 | -1.00 | 0.24 |  | Q56 | -1.70 | 1.23 |  | Q64 | -2.43 | 2.44 |  | K37 | -1.68 | 0.87 |  | F11 | -3.87 | 0.97 |
| Y34 | -3.67 | 0.59 |  | Y59 | -4.09 | 0.70 |  | R67 | -8.28 | 4.36 |  | M75 | -2.66 | 0.69 |  | K17 | -1.05 | 0.93 |
| N35 | -2.72 | 1.24 |  | I62 | -0.81 | 0.21 |  | E71 | -4.36 | 1.69 |  | A76 | -2.59 | 0.62 |  | E18 | -0.63 | 1.90 |
| L37 | -1.06 | 0.21 |  | L63 | -1.53 | 0.32 |  |  |  |  |  | G78 | -0.8 | 0.59 |  | L22 | -0.71 | 0.44 |
| M41 | -3.48 | 0.55 |  | L66 | -1.10 | 0.62 |  |  |  |  |  | P79 | -3.12 | 0.80 |  | K27 | -3.10 | 2.15 |
| L44 | -1.32 | 0.31 |  |  |  |  |  |  |  |  |  | L83 | -0.53 | 0.36 |  | R29 | -5.89 | 2.54 |
| K45 | -1.36 | 0.89 |  |  |  |  |  |  |  |  |  |  |  |  |  | Q30 | -3.72 | 1.88 |
| K48 | -0.60 | 0.19 |  |  |  |  |  |  |  |  |  |  |  |  |  | S34 | -2.69 | 1.13 |
| K51 | -0.58 | 0.03 |  |  |  |  |  |  |  |  |  |  |  |  |  | R37 | -4.58 | 1.89 |
| K53 | -2.17 | 1.62 |  |  |  |  |  |  |  |  |  |  |  |  |  | Q44 | -1.40 | 1.55 |
| R57 | -7.30 | 1.32 |  |  |  |  |  |  |  |  |  |  |  |  |  | K47 | -0.56 | 0.96 |
| K59 | -0.63 | 0.02 |  |  |  |  |  |  |  |  |  |  |  |  |  | F48 | -2.97 | 0.79 |
| F60 | -1.13 | 0.22 |  |  |  |  |  |  |  |  |  |  |  |  |  | K50 | -1.36 | 1.27 |
| L64 | -1.04 | 0.25 |  |  |  |  |  |  |  |  |  |  |  |  |  | R55 | -0.64 | 0.03 |
| R66 | -0.59 | 0.03 |  |  |  |  |  |  |  |  |  |  |  |  |  | R60 | -0.62 | 0.02 |
| K71 | -1.92 | 0.96 |  |  |  |  |  |  |  |  |  |  |  |  |  | R68 | -0.68 | 0.03 |
| K72 | -0.54 | 0.08 |  |  |  |  |  |  |  |  |  |  |  |  |  | K70 | -0.63 | 0.03 |
| A79 | -1.17 | 0.63 |  |  |  |  |  |  |  |  |  |  |  |  |  | K73 | -0.71 | 0.04 |
| A81 | -1.61 | 0.35 |  |  |  |  |  |  |  |  |  |  |  |  |  | R82 | -0.58 | 0.04 |
| K83 | -0.63 | 0.41 |  |  |  |  |  |  |  |  |  |  |  |  |  |  |  |  |

SD: standard deviation; *S. cerevisiae*: *Saccharomyces cerevisiae*; Heimdall_LC_3: Heimdallarchaeota_LC_3; Odin_LCB_4: Odinarchaeota_LCB_4; Thor_AB_25: Thorarchaeota_AB_25; Loki_GC14_75: Lokiarchaeum_GC14_75.
