# Supplementary material for: Coevolution of Eukaryote-like Vps4 and ESCRT-III Subunits in the Asgard Archaea
Source: mBio. 2020 May 19;11(3):e00417-20. doi: 10.1128/mBio.00417-20 (PMC7240154; doi:10.1128/mBio.00417-20)
Supplement: TABLE S4 [file mBio.00417-20-st004.docx]

**TABLE S4. The dominant amino acid residues of Vps4 Microtubule Interacting and Transport domain involved in binding with Vps20/32/60 are listed.**

| *S. cerevisiae* | | |  | Heimdall_LC_3 | | |  | Odin_LCB_4 | | |  | Thor_AB_25 | | |  | Loki_GC14_75 | | |
| --- | --- | --- | --- | --- | --- | --- | --- | --- | --- | --- | --- | --- | --- | --- | --- | --- | --- | --- |
| Residue | Total | SD |  | Residue | Total | SD |  | Residue | Total | SD |  | Residue | Total | SD |  | Residue | Total | SD |
| M1 | -0.63 | 1.44 |  | S2 | -3.14 | 2.07 |  | M4 | -0.67 | 0.56 |  | M1 | -0.63 | 0.09 |  | M1 | -3.14 | 1.20 |
| F6 | -3.73 | 0.51 |  | S3 | -2.45 | 2.67 |  | F9 | -0.56 | 0.40 |  | R4 | -0.65 | 0.05 |  | S6 | -1.06 | 0.34 |
| T8 | -0.86 | 0.28 |  | L8 | -0.51 | 0.29 |  | K17 | -0.74 | 0.57 |  | K10 | -0.64 | 0.04 |  | K7 | -5.83 | 1.39 |
| K9 | -4.72 | 2.29 |  | R27 | -1.51 | 2.43 |  | V20 | -1.78 | 0.50 |  | K15 | -0.54 | 0.02 |  | L8 | -2.44 | 0.43 |
| L13 | -1.73 | 0.39 |  | I32 | -0.65 | 0.21 |  | K21 | -0.55 | 0.05 |  | R22 | -0.50 | 0.02 |  | F11 | -1.51 | 0.67 |
| K16 | -0.64 | 0.40 |  | N33 | -1.04 | 1.14 |  | K24 | -2.38 | 1.68 |  | K25 | -0.57 | 0.02 |  | K15 | -0.86 | 0.09 |
| Y34 | -0.83 | 0.57 |  | Y34 | -1.18 | 1.08 |  | S52 | -0.70 | 0.89 |  | K26 | -0.53 | 0.02 |  | K17 | -0.81 | 0.03 |
| Y39 | -4.85 | 1.57 |  | R37 | -9.85 | 1.75 |  | K53 | -1.47 | 0.83 |  | K34 | -0.59 | 0.02 |  | K27 | -0.55 | 0.02 |
| M41 | -4.39 | 0.49 |  | E70 | -3.64 | 1.35 |  | K55 | -0.97 | 1.15 |  | K37 | -0.68 | 0.03 |  | R29 | -0.54 | 0.02 |
| L42 | -3.40 | 0.40 |  | E77 | -1.57 | 1.09 |  | N56 | -3.19 | 1.02 |  | K41 | -0.71 | 0.04 |  | R37 | -0.85 | 0.65 |
| L44 | -0.78 | 0.23 |  |  |  |  |  | I57 | -3.15 | 0.99 |  | K52 | -1.01 | 0.06 |  | I41 | -1.25 | 0.54 |
| K45 | -3.99 | 1.36 |  |  |  |  |  | L59 | -0.64 | 0.34 |  | K53 | -1.09 | 0.05 |  | Q44 | -1.61 | 0.79 |
| K48 | -9.41 | 1.17 |  |  |  |  |  | K60 | -7.72 | 1.52 |  | R58 | -0.90 | 0.04 |  | K47 | -2.28 | 0.96 |
| P50 | -1.95 | 0.44 |  |  |  |  |  | T61 | -1.42 | 0.55 |  | R65 | -0.73 | 0.04 |  | F48 | -4.67 | 0.70 |
| K53 | -3.05 | 1.19 |  |  |  |  |  | Q64 | -0.84 | 1.07 |  | R67 | -0.79 | 0.02 |  | K50 | -5.84 | 2.51 |
| R57 | -4.56 | 2.78 |  |  |  |  |  | Y65 | -1.99 | 0.71 |  | K73 | -0.66 | 0.02 |  | R55 | -5.73 | 1.39 |
| F60 | -0.72 | 0.34 |  |  |  |  |  | R68 | -1.80 | 0.36 |  | K74 | -0.54 | 0.02 |  | R60 | -0.89 | 0.05 |
| L64 | -0.72 | 0.25 |  |  |  |  |  |  |  |  |  | K88 | -0.78 | 0.02 |  | R68 | -0.70 | 0.06 |
|  |  |  |  |  |  |  |  |  |  |  |  |  |  |  |  | K70 | -0.70 | 0.05 |
|  |  |  |  |  |  |  |  |  |  |  |  |  |  |  |  | K73 | -0.63 | 0.04 |

SD: standard deviation; *S. cerevisiae*: *Saccharomyces cerevisiae*; Heimdall_LC_3: Heimdallarchaeota_LC_3; Odin_LCB_4: Odinarchaeota_LCB_4; Thor_AB_25: Thorarchaeota_AB_25; Loki_GC14_75: Lokiarchaeum_GC14_75.
